# High-efficiency enrichment enables identification of aptamers to circulating *Plasmodium falciparum*-infected erythrocytes

**DOI:** 10.1038/s41598-020-66537-1

**Published:** 2020-06-16

**Authors:** Eugene K. Oteng, Wenjuan Gu, Maureen McKeague

**Affiliations:** 1 Laboratory of Malaria and Vector Research, National Institute of Allergy and Infectious Diseases, National Institutes of Health, Rockville, Maryland 20852 USA; 2 Clinical Research Directorate/Clinical Monitoring Research Program, Leidos Biomedical Research, Inc., Frederick National Laboratory for Cancer Research, Frederick, Maryland 21702 USA; 3 Department of Pharmacology and Therapeutics, McGill University, 3655 Prom. Sir-William-Osler, Montreal, Quebec H3G 1Y6 Canada; 4 Department of Chemistry, McGill University, 801 Sherbrooke Street West, Montreal, Quebec H3A 0B8 Canada

**Keywords:** DNA, Bioanalytical chemistry

## Abstract

*Plasmodium falciparum* is the causative agent of the deadliest human malaria. New molecules are needed that can specifically bind to erythrocytes that are infected with *P. falciparum* for diagnostic purposes, to disrupt host-parasite interactions, or to deliver chemotherapeutics. Aptamer technology has the potential to revolutionize biological diagnostics and therapeutics; however, broad adoption is hindered by the high failure rate of the systematic evolution of ligands by exponential enrichment (SELEX). Here we performed parallel SELEX experiments to compare the impact of two different methods for single-strand recovery on the efficiency of aptamer enrichment. Our experimental results and analysis of SELEX publications spanning 13 years implicate the alkaline denaturation step as a significant cause for inefficient aptamer selection. Thus, we applied an exonuclease single-strand recovery step in our SELEX to direct aptamers to the surface of erythrocytes infected with *P. falciparum*. The selected aptamers bind with high affinity (low nanomolar K_d_ values) and selectivity to exposed surface proteins of both laboratory parasite strains as well isolates from patients in Asia and Africa with clinical malaria. The results obtained in this study potentially open new approaches to malaria diagnosis and surveillance.

## Introduction

Malaria is a global health problem, with 228 million clinical episodes and an estimated 405,000 deaths each year^1^. *Plasmodium falciparum* is the causative agent of the deadliest human malaria. A constellation of parasite-encoded proteins is displayed on the surface of infected erythrocytes (IEs) that are important in the life cycle of the parasite and mediate critical host-parasite interactions. One important host-parasite interaction is IE sequestration in the microvasculature which is thought to lead to the most severe manifestations of the disease. Therefore, ligand-binding molecules that can specifically identify and bind to IEs may disrupt important host-parasite interactions, deliver chemotherapeutics to parasitized cells, and have potential to further our understanding of the diversity of proteins expressed on the IE surface^2–5^.

Indeed, antibodies targeting IEs disrupt pathogenesis and contribute to naturally-acquired immunity to malaria^6–9^. Aptamers are an alternative to antibodies that may sufficiently serve as molecular recognition agents of IEs. Several aptamers have been selected that bind to targets and biomarkers of malaria infection (see review^10^ for examples). Aptamers are single-stranded nucleic acids that bind target molecules with antibody-like affinity and specificity through shape complementarity^11,12^. However, the nucleic acid composition of aptamers presents several advantages over antibodies including increased stability, cell-free synthesis (either by PCR or chemical synthesis), and simple modification for detection and immobilization. Aptamers have been developed against small molecules, peptides, proteins, and whole cells including bacteria and parasites^3,13–16^. As such, aptamers have proved useful for analytical applications, biosensors, drug delivery, potential drug substitutes, and large-scale biomarker discovery^17–19^.

Developing new aptamers requires the use of an *in vitro* procedure termed Systematic Evolution of Ligands by Exponential enrichment (SELEX). SELEX involves iterative rounds of targeted selection and PCR amplification followed by a single-strand recovery step^20,21^. Despite their tremendous potential, aptamers and SELEX have not reached the level of adoption expected for a technology developed almost 30 years ago. As such, antibodies remain the gold standard for molecular characterization and few aptamers have reached clinical significance^22^. One potential reason for the lack of aptamers in common use is because SELEX suffers from failure rate estimated to be as high as 70%^19,23^. Several recent improvements to the SELEX procedure including the use of specialized or massively parallel partitioning equipment, selections based on expanded genetic libraries, and modeling environmental parameters, have demonstrated promise in improving the aptamer success rate^19,24–28^. Despite the promise of these elegant studies, in general, these approaches require technology and expertise that is largely unavailable to most researchers. Thus, generic DNA SELEX remains by far the most popular aptamer isolation technique^29,30^. Efforts to identify which steps may contribute to the failure of SELEX are hampered by the complex dynamics of aptamer enrichment and the dearth of deep sequencing information from published studies to inform selections^31,32^. The current struggle to standardize the methodology, including use of different starting libraries, varying targets, partitioning methods, and buffers, further confound comparison between SELEX experiments. For example, a variety of methods have been employed for the single-strand recovery step following PCR, including asymmetric PCR, lambda exonuclease digestion, and magnetic separation with streptavidin-coated beads^33–35^. Each method has some degree of inefficiency but it is unclear whether these inefficiencies impact the outcome of SELEX experiments^33,36,37^. In particular, there has been no work to experimentally compare their relative effectiveness in the context of *de novo* aptamer selection.

Given the ongoing challenges of SELEX and its high failure rate, we performed parallel selection experiments towards *Plasmodium falciparum* IEs coupled with high-throughput sequencing to directly compare the impact of the single-strand recovery step on aptamer enrichment for the first time. We demonstrate that the commonly used alkaline denaturation-based method fails to enrich aptamers under conditions where an alternative strand separation, lambda exonuclease digestion, succeeds. To support these findings, we analyzed SELEX publications spanning a 13-year period and found a significant association between SELEX experiments that required a high number of iterative selection rounds and the choice of alkaline denaturation as the single-strand recovery method. Taken together, these results implicate the alkaline denaturation step as a possible cause for inefficient aptamer selection and thus potential SELEX failure.

In our comparative study, we sought to isolate aptamers capable of recognizing the surface of IEs. Due to the incorporation of a rigorous strand separation method, we efficiently isolated a panel of high affinity aptamers and identified an aptamer that recognized both laboratory parasite strains as well as recently adapted isolates from patients with clinical malaria. We hypothesise that the target of this aptamer is likely a parasite-induced IE membrane protein common between parasites from geographically distinct areas. We expect that characterization of the target molecules of the isolated aptamers will aid in further defining the IE surface and global distribution of potential drug and vaccine targets, a new exciting application for aptamer technology.

## Results

### Parallel SELEX methods were used to compare the impact of the single-strand recovery step

**Figure 1:**
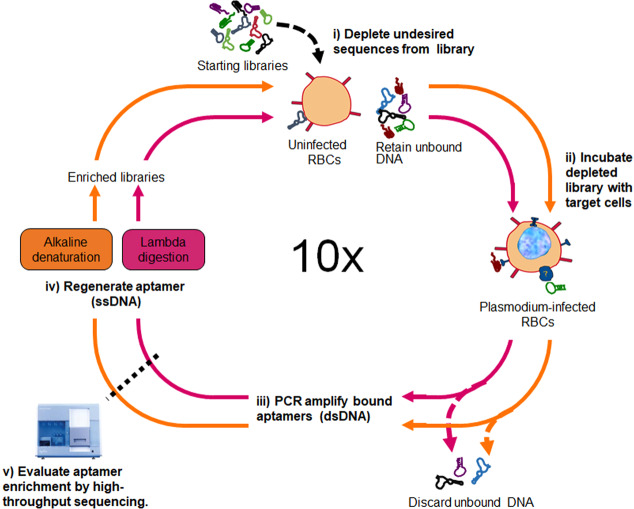
A parallel SELEX scheme was employed to select aptamers to *P. falciparum*-infected red blood cells (RBCs). (i) Combinatorial ssDNA libraries, consisting of oligonucleotides with a centrally randomized region of 45 nucleotides flanked by specific primers, are removed of sequences that bind to uninfected RBCs in a negative selection step. (ii) The depleted libraries are incubated with *P. falciparum*-infected RBCs to enforce selection of aptamers binding determinants unique to infected erythrocytes (IEs). (iii) Following thorough washing, bound sequences are amplified by PCR. (iv) Aptamers are regenerated as ssDNA either by alkaline denaturation or digestion with lambda exonuclease. The resulting enriched ssDNA pools are used in the next round of the negative selection, positive selection, and amplification. In the 10^th^ SELEX round, the selected ssDNA from the two independent selections are analyzed for aptamer enrichment using the Illumina high throughput sequence platform.

Two SELEX experiments were performed in parallel, both starting from a library of 10^14^ ssDNA (Fig. 1), to test the impact of the single-strand recovery step. The SELEX process has been extensively described elsewhere^38^; in brief, a negative selection was performed by incubating the starting libraries with uninfected erythrocytes. Next, the libraries depleted of sequences that bind to uninfected erythrocytes were subjected to a positive selection by incubating the remaining sequences with IEs. Sequences with weak or no binding to the IEs were removed with several washing steps and discarded. Putative aptamer sequences were isolated and amplified by PCR. Following PCR, for the single-strand aptamer recovery method, we used either lambda exonuclease digestion (lambda) or immobilization of biotinylated dsDNA onto streptavidin-coated beads followed by denaturation of dsDNA by alkaline treatment (alkaline). The single stranded sequences from these parallel methods resulted in two enriched libraries for use in the following parallel rounds of SELEX.

### High-throughput sequencing and bioinformatics analysis reveal a greater sequence enrichment using the lambda exonuclease single-strand recovery method

**Figure 2:**
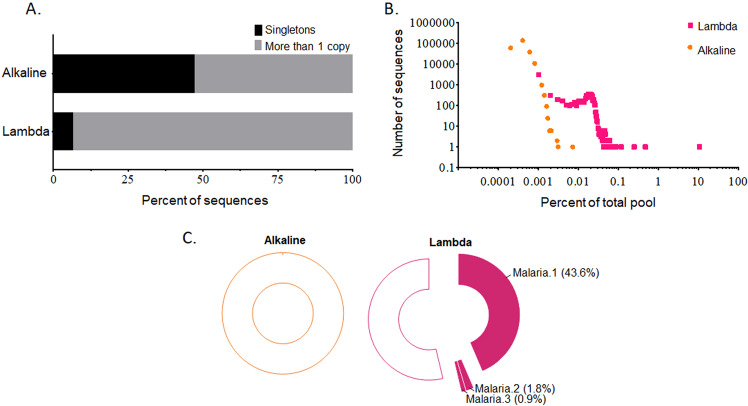
High-throughput sequencing and bioinformatics analysis of the SELEX enrichment. (**A**) Based on the percent of individual copies of unique sequences (singletons), sequencing results reveal a reduced enrichment using the alkaline-based SELEX compared with a parallel experiment performed using lambda exonuclease as the ssDNA recovery method. (**B**) The plot of number of different sequences as a function of enrichment. (**C**) Representative doughnut graphs of primary sequence alignments. The top 500 unique sequences shows no aptamer clustering from the alkaline SELEX whereas the lambda SELEX reveals a prominent aptamer cluster represented by **Malaria.1**. Two other clusters, represented by **Malaria.2** and **Malaria.3** were also identified. Open regions represent sequences that could not be clustered.

Following 10 rounds of SELEX, the aptamer pools were sequenced using the Illumina platform and analyzed using R software. In the alkaline SELEX method, 47.1% of sequences were present in the library more than once, meaning that 52.9% of the sequences were singletons (Fig. 2A). In contrast, lambda SELEX showed much greater enrichment with only 6.7% of the pool remaining singletons (Fig. 2A). Within the alkaline SELEX final pool, the most frequent sequence occurred at a frequency of 0.004% (37 times in 965,748 sequencing reads). In comparison, one sequence, **Malaria.1**, was present at a frequency of 10.7% (40,640 times in 378,439 reads) of the lambda SELEX final pool (Fig. 2B, and Table S1 in the Supplementary).

SELEX is expected to co-enrich sequences that fold into similar target binding shapes or with similar function; therefore, to further test enrichment, we clustered the top 500 unique sequences from each pool by primary sequence homology using Clustal Omega. As shown in Fig. 2C, none of the analyzed sequences within the alkaline SELEX showed significant homology and could not be clustered. In the lambda SELEX, however, a large aptamer cluster comprising 43.6% of the total analyzed sequences was readily identified. Not surprisingly, this cluster contained the most frequently observed individual sequence, **Malaria.1**. Two other clustered populations of sequences comprising 1.8% and 0.9% of the total analyzed sequences were also readily identified. These populations were represented by the frequent sequences **Malaria.2** and **Malaria.3**, respectively.

Our data suggest that the single-strand recovery step is a key determinant in the course of SELEX. In particular, the sequencing results show that the alkaline method fails to efficiently enrich aptamers whereas under identical conditions, lambda digestion is successful. Other research groups have reported that free streptavidin and biotinylated antisense strands are released from streptavidin beads using the alkaline method which could anneal to the aptamers. They have speculated that this could have a dampening effect on aptamer enrichment despite the application of effective selective pressure^33–35,39^. Consistent with this, **Malaria.3** was the top sequence recovered from alkaline SELEX but observed at a 64.7 times lower frequency than observed in lambda SELEX (Table S1 in the Supplementary). Likewise, **Malaria.2** was found at a frequency 759.6 times lower in the alkaline SELEX when compared with lambda SELEX. Interestingly, **Malaria.1** was not identified in the alkaline SELEX final pool.

### Literature analysis confirms that poor enrichment is correlated with the alkaline single-strand recovery method

Intrigued by the difference in enrichment between lambda and alkaline SELEX, we examined whether our observations were representative of SELEX experiments performed by other groups against unrelated targets. To assess this, we queried the data from a previous aptamer database (AptamerBase, https://github.com/micheldumontier/aptamerbase)^40^. Publications used for analysis were selected as described in Supplementary Table S2 and the curated data is available in Supplementary Table S4. Given that enrichment is rarely reported in past SELEX literature, we used the number of SELEX rounds as a surrogate measure of the efficiency of aptamer enrichment. We reasoned that studies requiring a higher number of SELEX rounds (≥15 rounds) reflect inefficient aptamer enrichment whereas efficient selection requires fewer rounds (≤10 rounds).

First we calculated the crude odds ratios for categorical variables and tested significance of the odds ratios using Fisher’s exact test. We found that the likelihood of experiencing inefficient enrichment in SELEX experiments using alkaline denaturation is 28.0 times that of those that did not use alkaline denaturation (p-value = 0.0002, Table S3 in the Supplementary). To ensure that the initial diversity of the library was not a confounding parameter in the analyses, we compared the size of the library and the length of the random region for experiments employing alkaline treatment against experiments using alternative single-strand generating methods and found no significant difference (Figs. S1 and S2 the Supplementary). Furthermore, multivariate analysis accounting for the type of aptamer target, method used to partition aptamers from non-binding sequences, binding affinity of recovered aptamers and the year of publication, showed the likelihood of inefficient enrichment in experiments using alkaline denaturation is 34.5 times that of studies that do not use the alkaline method (p-value = 0.0041, 95% CI 3.1–382.8, Table 1).

**Table 1:** Multivariate logistic regression for successful SELEX enrichment.

Characteristics	Odds Ratio* (95% Confidence Intervals)	p-value
Alkaline use	0.029 (0.003 to 0.327)	0.0041
Log-average K_d_	0.541 (0.165 to 1.777)	0.3114
Target type: Protein vs. non-protein	2.362 (0.206 to 27.124)	0.4901
Partitioning method: Affinity chromatography vs. other	5.638 (0.762 to 41.706)	0.0903
Year	1.037 (0.883 to 1.219)	0.6554

*Odds ratios are adjusted for alkaline use, log-average K_d_, target types, partitioning method and year of publication.

Simple univariate analysis (see Supplementary Table S3) indicates that cell targets and employing a centrifugation-based partitioning method may be less efficient for enrichment than other strategies. These factors are often constrained by practicality, further highlighting the importance of selecting an efficient non-alkaline method when design a SELEX experiment. Interestingly, our results also show that there is no significant (p-value = 0.3114, Table 1) correlation between the number of rounds used in SELEX and the average affinity of the aptamer (measured as the logK_d_). The lack of correlation between number of rounds and affinity is consistent with our previous work^29^ and suggests that increasing the number of rounds of SELEX does not lead to better aptamers^41^, and could be a potential surrogate for an inefficient enrichment.

### Highest frequency aptamer sequences bind to infected erythrocytes

**Figure 3:**
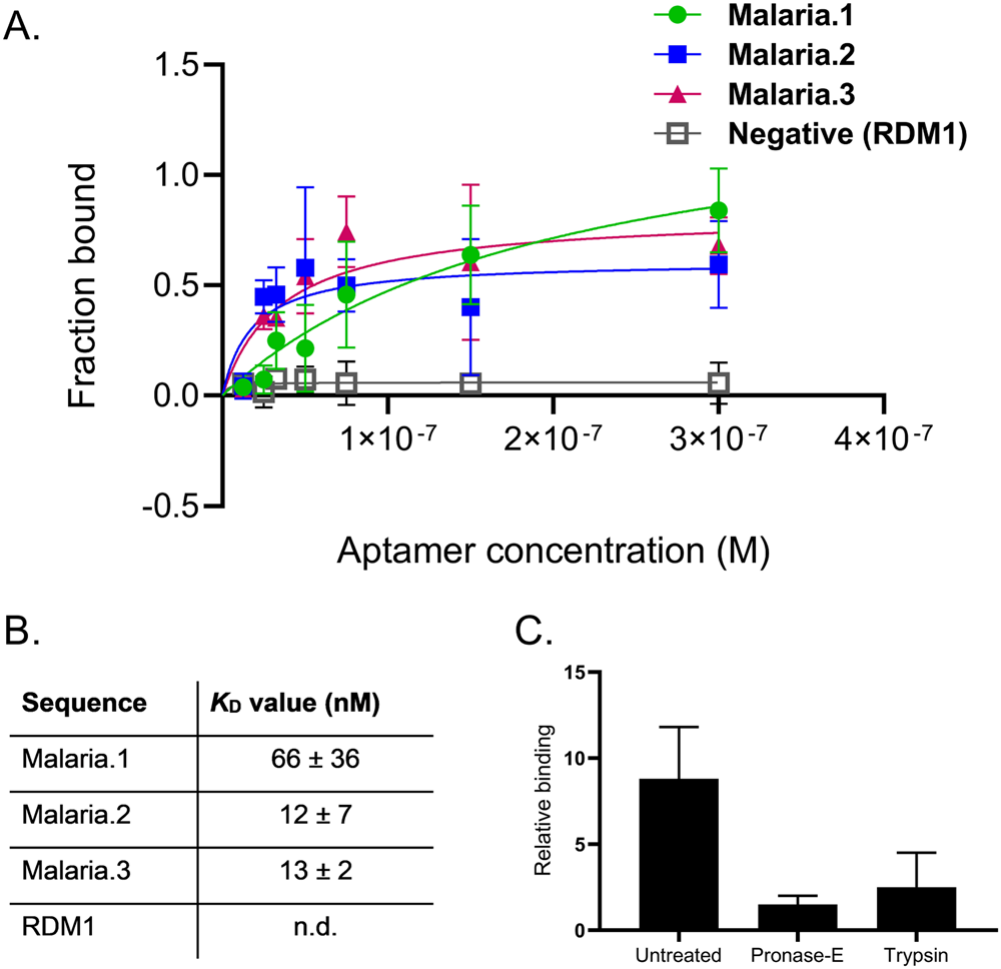
Binding characteristics of selected aptamers to infected erythrocytes (IEs). (**A**) Binding isotherm: Putative aptamer sequences were labeled at the 5′ end with [^32^P]. Five million cells per well were incubated with increasing concentrations of labeled sequences in buffer. After three washes, the amount of ^32^P-labeled aptamer associated with cells was determined by liquid scintillation counting. Data were normalized by fraction bound. **Malaria.1, Malaria.2** and **Malaria.3** bind to IEs with high affinity but the randomly generated control sequence of the same length (**RDM1**) shows no binding to IEs. (**B**) *K*_D_ values were determined using a one-site specific binding analysis in GraphPad Prism. *K*_D_ for each sequence is reported as the mean ± standard deviation from 3 assays performed in duplicate. (**C**) Five million cells per well of IEs were pre-treated with extracellular proteases. 150 nM of labeled **Malaria.1** or labeled **RDM1** was incubated with the cells. After three washes, the amount of ^32^P-labeled aptamer was determined by liquid scintillation counting. Fold change in binding of aptamer **Malaria.1** was determined by normalizing to the background binding. Background binding was determined as the average signal of two random sequence oligonucleotides (**RDM1** and **RDM2**) to the cells. The reduced binding of **Malaria.1** when treated with Pronase and Trypsin (6 fold and 5 fold respectively) suggest the aptamer binds to an IE membrane protein. Data shown as mean ± SEM, n = 3 assays performed in duplicate.

Next, we individually synthesized and validated the binding of **Malaria.1**, **Malaria.2**, **Malaria.3**, the highest frequency sequences (Supplementary Table S1) from the three aptamer clusters isolated by lambda SELEX. All putative aptamer sequences were truncated to their variable regions and radiolabeled at the 5′ end with [^32^P] to enhance synthesis efficiency and ease of detection. Five million cells per well were incubated with increasing concentrations of labeled sequences in buffer. After three washes, the amount of ^32^P-labeled aptamer associated with cells was determined by liquid scintillation counting. Data were normalized by fraction bound. The binding affinities of the selected aptamers to IEs were then determined by evaluating the apparent dissociation constants. **Malaria.1** displayed binding toward IEs in the nanomolar range (apparent K_d_ ~60 nM). Given recent concerns of the functionality of selected aptamers^42^, we confirmed that the apparent binding is a result of a specific aptamer interaction by performing the binding assay using a random DNA oligonucleotide sequence of the same length, **RDM1**. **RDM1** showed minimal binding to IEs up to concentrations of 300 nM (Fig. 3A). Interestingly, both **Malaria.2** and **Malaria.3** showed apparent high affinity binding to IEs (apparent K_d_~ 12 and ~13 nM respectively) suggesting that the enrichment resulted in several high affinity binding aptamers (Fig. 3B). Interestingly, the highest frequency sequence, **Malaria.1**, was not the highest affinity sequence. Even though **Malaria.1** was present between 20 and 40 times higher in the final sequencing round, our analysis indicates an approximately 5-fold weaker binding than **Malaria.2** and **Malaria.3**. Several other aptamer selections have demonstrated that the most enriched sequences are not always the highest affinity aptamers^32,43^. As such, it is critical to couple efficient enrichment with an analysis of several candidates^44^. Nevertheless, given that our study focused on the importance of efficient enrichment, we performed detailed studies on the most enriched sequence, **Malaria.1**.

The saturated, high affinity binding of **Malaria.1** and other selected aptamers suggest they target cell surface determinants. To test whether **Malaria.1** was binding to proteins on the cell surface, we pre-treated IEs with the proteinases trypsin or pronase E to remove the likely targets and then added **Malaria.1** or the random sequence control, **RDM1**, to the cells. Figure 3C shows that treatment with extracellular proteases nearly abolishes **Malaria.1** binding to IEs, indicating that the binding partner of **Malaria.1** is likely an IE surface membrane protein and further supports a specific binding interaction between the selected aptamer and infected erythrocytes.

### Characterization of the broad spectrum binding of Malaria.1

**Figure 4:**
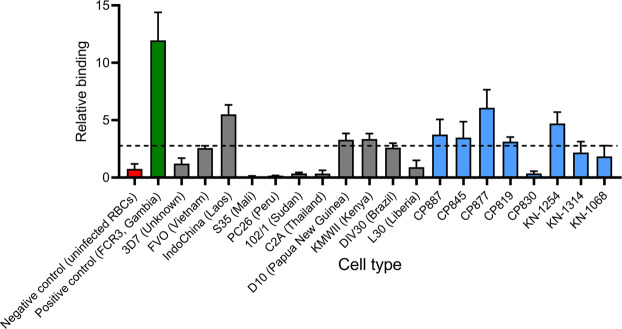
Relative binding of **Malaria.1** to various cell types. Five million cells for each strain per well were incubated with 150 nM of **Malaria.1**. After three washes, the amount of ^32^P-labeled aptamer was determined by liquid scintillation counting. Fold change in binding of aptamer **Malaria.1** was determined by normalizing to the background binding. Background binding to each cell type was determined as the average signal of two random sequence oligonucleotides (**RDM1** and **RDM2**). The negative control cells are uninfected RBCs (red). The positive control cell type is the strain used in the selection experiment, FCR3 (green). Bars in grey represent cultured cell lines. Bars in blue indicate cells from patients, where CP cell lines are short-term cultures derived from recent Cambodian infections and KN from Malian infections. The dashed horizontal line represents the of threshold of positive binding defined as the mean fold difference plus 3 standard deviations for random sequence binding to uninfected erythrocytes from 6 US donors. Values and error bars represent the mean and standard deviation of three replicates.

While only one *P. falciparum* isolate was used for SELEX, multiple *P. falciparum* strains can be found circulating malaria endemic areas each displaying a complex array of antigenically variable IE determinants^7,45,46^. Therefore, we next examined the breadth of **Malaria.1** binding by screening radiolabeled aptamers against a variety of cell types (Fig. 4). First, consistent with the incorporation of a negative selection step, we found that **Malaria.1** showed no affinity to uninfected erythrocytes. This indicates that the target of **Malaria.1** is generated or induced by *P. falciparum*. Next, we screened **Malaria.1** against a panel of laboratory-adapted *P. falciparum* isolates. We found that **Malaria.1** displayed somewhat broad binding, recognizing 4 out of 12 parasite lines tested including the *P. falciparum* isolate used as the target of SELEX (Fig. 4). Finally, we determined whether **Malaria.1** could bind newly adapted parasite lines isolated from patients symptomatic with malaria from Asia and Africa. The aptamer recognized parasites isolated from Cambodian (4 out of 5 tested) and Malian (1 out of 3 tested) patients presenting with clinical malaria (Fig. 4).

The different relative binding observed can conservatively attributed to different expression levels of a surface protein, a known phenomena in *Plasmodium* and often linked to immune evasion^47^. Taken together, these data suggest that **Malaria.1** likely binds to a parasite-induced IE surface protein commonly expressed during clinical malaria infections.

## Discussion

Nearly 30 years of aptamer research has demonstrated their feasibility as binding agents with a remarkable range of applications. Wide adoption of this technology, including the isolation of novel aptamers against important targets, is lagging because the aptamer selection process is complex and poorly understood. Here, we used the combination of high-throughput sequencing and parallel selection methods to isolate a panel of high affinity aptamers directed to the surface of *Plasmodium falciparum*-infected red blood cells. Our parallel SELEX design, coupled with deep sequencing analysis, allowed us to experimentally evaluate key steps in generic DNA SELEX leading to successful enrichment of target binding sequences. Importantly, we demonstrate that the single-strand recovery step is a critical determinant for successful aptamer enrichment. Specifically, we show that the alkaline method inefficiently enriches aptamers under conditions where enzymatic digestion is readily successful. Importantly, the inefficiency of alkaline-based aptamer enrichment was not easily bypassed with high-throughput sequencing of the final pool. Moreover, by analyzing published literature we found a strong association between use of the alkaline method and prolonged and inefficient aptamer selections performed by other groups. Multivariate analysis indicates that the alkaline method for single strand recovery is inefficient regardless of the type of target being selected against, the partitioning method used to separate aptamers from non-binding sequences, and the intrinsic binding affinity of the isolated aptamer against its selected target.

The ideal outcome of a SELEX experiment is a number of high frequency sequences identified which can be rapidly characterized for high affinity binding to the target of interest^44^. As such, a successful experiment must allow for the efficient enrichment of the putative aptamers well above background non-binding sequences^48^. Inefficient enrichment can make SELEX prohibitively resource and labor intensive because more rounds of selection are required. One possible drawback of requiring more rounds of selection is that less-structured non-binding sequences are more easily amplified by PCR polymerases, and thus may be enriched over the desired structure putative aptamers^41^. Moreover, when a pool of selected aptamers is evaluated by traditional low throughput sequencing, there is a high likelihood aptamers will not be identified. Results from our parallel selection highlight both the likelihood and importance of these phenomena. **Malaria.2** and **Malaria.3** were identified in the alkaline SELEX but were only enriched to very low levels, and may not have been identified at all if a traditional low-throughput sequencing method was used (e.g., Sanger sequencing). While SELEX experiments that have failed to enrich aptamers are rarely published, we can extrapolate from our study that use of the alkaline method is not only a determinant of inefficient SELEX but may also be a factor for the historically high failure rate of SELEX^49^.

There are many emerging strategies to improve the success of SELEX experiments such as performing only one or a few rounds of SELEX with the help of improved partitioning^41,48^. These strategies have been successful where capillary electrophoresis or prior knowledge of the specific target is available and accessible. Our results lead us to recommend evaluating aptamer enrichment with high-throughput sequencing and to carefully consider the single-strand recovery step when designing a SELEX experiment to isolate aptamers *de novo*. Our work indicates that alternative strand isolation methods including lambda digestion offer comparable yields and improved ease of use compared to alkaline denaturation^34,50^. Importantly, we expect that use of the lambda-based method should enrich putative aptamer candidates using fewer rounds of selection leading to increased success of most commonly used SELEX protocols. Establishment of an easily accessible, reliable method to isolate high affinity aptamers should result in a wider adoption of aptamer biotechnology.

It is interesting that only three aptamer clusters were identified despite employing the more efficient lambda SELEX and screening the complete IE surface proteome. One might expect enrichment of multiple aptamers against the diverse range of predicted parasite-encoded exported targets because as long as the binding of one aptamer does not interfere with the binding of another aptamer, selections are highly multiplexed against complex targets^31,51^. In contrast, our findings suggest that either a limited number of IE determinants are displayed or are accessible to aptamers during SELEX. Definitive identification of the aptamer target(s) will be useful to better understand the diversity of determinants displayed on the IE surface.

**Malaria.1** was identified as the most abundant sequence from the lambda SELEX. Interestingly, this was not the highest affinity sequence recovered. While this is a common phenomenon with SELEX, it may be that the primer binding regions of **Malaria.1**, which were truncated for the analysis, were required. Indeed, nucleotides in the primer regions are occasionally involved in binding, but often they have little or no effect^30^. Nevertheless, the **Malaria.1** sequence tested has several characteristics of a “good” aptamer. First, **Malaria.1** has no measurable affinity to uninfected erythrocytes, likely due to the negative selection step employed (Fig. 1). Second, **Malaria.1** recognizes multiple laboratory isolates and could bind to clinical malaria infections from at least two different parts of the world. This finding was surprising given that SELEX typically results in an aptamer with high specificity; isolating an aptamer with broad-spectrum binding generally requires a deliberate poly-target selection strategy^52,53^. Therefore, it is more likely that **Malaria.1** binds to a specific IE determinant that is common between geographically disparate circulating strains represented by the laboratory and clinical isolates tested. Further work will examine whether the target is a glycoprotein like those critical to the lifecycle of Plasmodium^54–57^. Importanly, **Malaria.1** displays an apparent affinity in the nanomolar range and therefore be suitable for definitive target identification via pull down and mass spectroscopy.

There is a critical need for a better understanding of malaria as well as tests for the rapid diagnosis and surveillance of malaria. As such, there have been many reports describing the selection of new aptamers^3,4,11,58–61^ to malaria targets as well as novel aptamer-based biosensors^62–64^. Previous studies generating aptamer probes to the IE surface have used pre-defined *Plasmodium* recombinant protein fragments or intact IEs and sophisticated microfluidic fabrication. In contrast, our work describes a highly accessible generic DNA SELEX that permits the successful identification of anti-*Plasmodium* aptamers *de novo*. Our approach does not require *a priori* knowledge of the surface proteome of IEs. By adjusting the positive and negative selection steps of SELEX, the method we describe here may be used for studies to probe the unique surfaceome of IEs mediating the various manifestations of malaria including cerebral malaria and pregnancy-associated malaria. In particular, future studies will use the isolated aptamers here, as well as additional aptamer selections, for aptamer-facilitated biomarker discovery (AptaBiD)^65,66^ to identify and discover potential structurally-conserved proteins displayed on the surface of erythrocytes parasitized with *Plasmodium falciparum*.

## Materials and Methods

### ***P. falciparum*** culture

*P. falciparum* strains were grown under standard conditions^67^. Laboratory lines were confirmed negative for *Mycoplasma* contamination using the Mycotrace Detection Kit (PAA Laboratories, Pasching, Austria). Clinical isolates were derived from patients presenting with symptoms of uncomplicated clinical malaria. The Ethics Committee of the Faculty of Medicine, Pharmacy and Odontostomatology at the University of Bamako, the Cambodian National Ethics Committee for Health Research, and the National Institute of Allergy and Infectious Diseases Institutional Review Board approved protocol activities and all experiments were performed in accordance with their relevant guidelines and regulations. All adults or the parents or guardians of children provided written informed consent. The protocols are registered at Clinicaltrials.gov (NCT00669084 and NCT01240603). Details of the collection have been previously described^68,69^.

### SELEX library, primers and control sequences

DNA library and PCR primers were purchased from Integrated DNA Technologies (Coralville, IA). The library used was identical in template to one previously validated for cell surface binding^70^. The library contained a central randomized sequence of 45 random nucleotides flanked by 20 nucleotide primer hybridization sites (5′**-**ACGCTCGGATGC-CACTACAG-45N-CTCATGGACGTGCTGGTGAC-3′). Unmodified forward primer was used in all PCR steps (5′-ACGCTCGGATGCCACTACAG-3′). The reverse primer (5′**-**GTCACCAGCACGTCCATGAG-3′) was either biotin labeled at the 5′ end to enable the binding of double-stranded DNA to streptavidin-coated magnetic beads or modified with 5′ phosphate to facilitate strand selective digestion by lambda exonuclease. Two randomly generated 45 nucleotide oligomers were used as aptamer controls (**RDM1**, GCAACCAGGCAGACTTGGCGGTAGGTCCTAGTGCAGCGGGTTGGG; **RDM2**, CGACGTTCTAAGCGTTGGACGGTGTGAATCGCGACCCAGGTCTTC).

### Strand separation

For alkaline denaturation-based SELEX, the strand separation after PCR was performed with streptavidin-coated magnetic beads (M-270 Dynabeads, Invitrogen, Carlsbad, CA) according to manufacturer’s instructions. This brand was chosen because many groups working with SELEX commonly use these beads. Briefly, PCR product was incubated for 25 minutes on a rotator with 5 mg beads. After 2 washes with binding and washing buffer (10 mM Tris–HCl pH 7.5, 1 mM EDTA, 0.2 M NaCl) an alkaline denaturation was performed with 500 μL freshly prepared 200 mM NaOH to melt the DNA. The tubes were applied to a magnet and the eluted ssDNA was desalted with a Sephadex G-25 column (NAP-5, GE Healthcare, UK). In the lambda SELEX experiment, PCR products featuring a phosphorylated antisense strand were treated with 5 units of lambda exonuclease for 30 min at 37 °C to digest the antisense strand. The regenerated single-stranded aptamers were isolated by ethanol precipitation for the next round of counter-selection, selection, and amplification.

### SELEX

Two SELEX experiments were performed in parallel to determine the effect of strand separation methods on aptamer enrichment. For a typical round of SELEX, ssDNA recovered from the previous round was dissolved in 200 μL Aptamer Binding buffer [AB buffer, RPMI-1640 (pH 7.2) supplemented with BSA (1 mg/mL, Sigma, St Louis, MO), yeast tRNA (0.1 mg/mL, Invitrogen), sheared salmon sperm DNA (0.1 mg/mL, Invitrogen)] and then denatured by heating at 95 °C for 5 min and cooling to 4 °C before binding. To avoid selecting for aptamers specifically recognizing uninfected erythrocytes, the library was incubated with 5 × 10^6^ uninfected erythrocytes for 30 min at room temperature and following centrifugation, unbound sequences were recovered for the selection phase. The sequences were then incubated with 10^6^ Percoll-purified *P. falciparum*-infected erythrocytes (FCR3 strain) for 10 min at room temperature. After washing in AB buffer, infected erythrocytes were lysed in 100 μL 95 °C water, debris was pelleted, and 30% of the resultant supernatant was amplified by PCR. PCR Supermix 1X Ex Taq Version 2.0 (Takara, Mountain View, CA), unmodified forward and reverse primers modified with either 5′ biotin or phosphate (0.2 μM final concentration) were added to bring the reaction to 100 μL final volume. Reactions were amplified for 13–20 cycles of 0.5 min at 94 °C, 0.5 min at 56 °C, and 0.5 min at 72 °C, followed by 5 min at 72 °C. Strand separation using lambda exonuclease digestion or streptavidin immobilization followed by alkaline denaturation was used to regenerate ssDNA to use in the next round of SELEX.

For the first round of either selection experiment, the amount of naive ssDNA library was 10 nmol dissolved in 1 mL of AB buffer and the counter-selection step was eliminated. Following 10 rounds, the resultant pool was PCR amplified with unmodified primers for analysis using the Illumina high-throughput sequencing platform (Illumina, San Diego, CA).

### High-throughput sequencing

Aptamer libraries were constructed using the Illumina Multiplexing Sample Preparation Oligonucleotide Kit (PE-400–1001, Illumina) with the exception that the DNA was not sheared. Libraries were size-selected to remove unincorporated adapters on a 2% NuSieve Agarose gel (Lonza, Basel, Switzerland), stained with SybrGold (Invitrogen), and visualized on a Dark Reader (Clare Chemical, Dolores, CO). To insure that the library was not over amplified, test amplification was performed where aliquots of a PCR reaction were removed every 2 cycles from cycles 4 through16. These aliquots were evaluated on a 2% agarose gel and an optimal cycle number was selected for a subsequent large-scale amplification. Final libraries were quantitated using qPCR and pooled in an equimolar ratio for sequencing. Paired-end 100 base reads were obtained on a GA_ii_x using version 4 chemistry (Illumina).

Multiplexing yielded ~2 M reads per sample. See Supplementary for sequencing files. Sequences were filtered to have an exact match of the primers and a matching multiplexing tag. Only the randomized regions were used for further analysis. Identical sequences were collated using an R-script^71^. Unique sequences were grouped according to primary sequence similarity using Clustal Omega (http://www.ebi.ac.uk, EMBL-EBL).

### Cell binding assay

Binding of individual aptamers to cells was performed in 96-well DNA Lo-Bind Deepwell plates (Eppendorf, Hauppauge NY) in triplicate with 5′ [^32^P]-labeled DNA. Five million cells per well were incubated with 150 nM DNA in 25 μL AB buffer. After three washes in 500 μL AB buffer, cells were fixed in 1% paraformaldehyde and then lysed in deionized water. The amount of ^32^P-labeled aptamer associated with cells was determined by liquid scintillation counting. The average binding intensity of **RDM1** and **RDM2** was used to determine the level of background binding to each cell type. The apparent equilibrium dissociation constant (K_d_) of the aptamer–cell interaction were obtained by fitting using one-site specific binding in Prism version 8.0 (GraphPad Software).

### Protease susceptibility studies

Infected mid- and late-state IEs (24–36 hours post invasion) were washed twice in PBS and incubated at 5% hematocrit with 1 mg/mL Pronase E from *Streptomyces griseus* (Sigma) in PBS supplemented with 1 mM CaCl_2_ and 1 mM MgCl_2_ for 30 min at 37 °C or 1 μg/mL of porcine-modified trypsin (Sigma) in PBS for 30 min at 37 °C. After extensive washing with AB buffer supplemented with complete Proteinase Inhibitor Cocktail (Roche, Basel, Switzerland), the treated cells were used for aptamer-binding assays.

### Aptamer literature search

42 publications were analyzed from literature described in AptamerBase and publications in AptamerBase were curated as described previously^29,40^. To compare previous SELEX literature to our experimental data, we only analyzed literature describing generic SELEX methods towards cell and protein-based targets using DNA templates. The eligibility requirements for the literature used in our analysis are outlined in the Supplementary Table S2, and curated data is available in Supplementary Table S4.

### Statistical analysis

We used Fisher’s exact test for categorical variables and Wilcoxon’s Rank-Sum test for continuous variables to investigate the crude associations between risk factors and prolonged amplification of aptamers. Multivariate logistic models were used to study the adjusted associations. Inefficient amplification was defined as SELEX completed at ≥15 rounds whereas efficient amplification was defined as SELEX completed at ≤10 rounds. The potential risk factors we examined were alkaline denaturation use, average apparent K_d_, target type, partitioning method, and year of publication. We calculated the crude odds ratios for categorical variables and tested significance of the odds ratios using Fisher’s exact test. The medians of continuous factors were calculated by inefficient versus efficient amplification and compared using the Wilcoxon test.

## Supplementary information

Supplementary information.

Supplementary information 2.

Supplementary information 3.

Supplementary information 4.

## References

[1] WHO (2016) *World Malaria Report*.

[2] Eda K, Eda S, Sherman IW (2004). Identification of peptides targeting the surface of Plasmodium falciparum-infected erythrocytes using a phage display peptide library. Am. J. Trop. Med. Hyg..

[3] Barfod A, Persson T, Lindh J (2009). *In vitro* selection of RNA aptamers against a conserved region of the Plasmodium falciparum erythrocyte membrane protein 1. Parasitol. Res..

[4] Birch CM, Hou HW, Han J, Niles JC (2015). Identification of malaria parasite-infected red blood cell surface aptamers by inertial microfluidic SELEX (I-SELEX). Sci. Rep..

[5] Zhang L (2017). Molecular Elucidation of Disease Biomarkers at the Interface of Chemistry and Biology. J. Am. Chem. Soc..

[6] Chan JA (2012). Targets of antibodies against Plasmodium falciparum-infected erythrocytes in malaria immunity. J. Clin. Invest..

[7] Bull PC (1998). Parasite antigens on the infected red cell surface are targets for naturally acquired immunity to malaria. Nat. Med..

[8] Williams AR (2012). Enhancing blockade of Plasmodium falciparum erythrocyte invasion: assessing combinations of antibodies against PfRH5 and other merozoite antigens. PLoS Pathog..

[9] Miura K (2013). Relationship between malaria incidence and IgG levels to Plasmodium falciparum merozoite antigens in Malian children: impact of hemoglobins S and C. PLoS One.

[10] Nik Kamarudin, N. A. A., Mohammed, N. A. & Mustaffa, K. M. F. Aptamer Technology: Adjunct Therapy for Malaria. *Biomedicines* **5**, 10.3390/biomedicines5010001 (2017). 10.3390/biomedicines5010001 PMC5423489 28536344

[11] Cheung YW (2013). Structural basis for discriminatory recognition of Plasmodium lactate dehydrogenase by a DNA aptamer. Proc. Natl Acad. Sci. USA.

[12] Kato K (2016). Structural basis for specific inhibition of Autotaxin by a DNA aptamer. Nat. Struct. Mol. Biol..

[13] McKeague M, Derosa MC (2012). Challenges and opportunities for small molecule aptamer development. J. Nucleic Acids.

[14] Shangguan D (2006). Aptamers evolved from live cells as effective molecular probes for cancer study. Proc. Natl Acad. Sci. USA.

[15] Homann M, Lorger M, Engstler M, Zacharias M, Goringer HU (2006). Serum-stable RNA aptamers to an invariant surface domain of live African trypanosomes. Comb. Chem. High. Throughput Screen..

[16] Morris KN, Jensen KB, Julin CM, Weil M, Gold L (1998). High affinity ligands from *in vitro* selection: complex targets. Proc. Natl Acad. Sci. USA.

[17] Keefe AD, Pai S, Ellington A (2010). Aptamers as therapeutics. Nat. Rev. Drug. Discov..

[18] Hathout Y (2015). Large-scale serum protein biomarker discovery in Duchenne muscular dystrophy. Proc. Natl Acad. Sci. USA.

[19] Gold L (2010). Aptamer-based multiplexed proteomic technology for biomarker discovery. PLoS One.

[20] Tuerk C, Gold L (1990). Systematic evolution of ligands by exponential enrichment: RNA ligands to bacteriophage T4 DNA polymerase. Science.

[21] Ellington AD, Szostak JW (1990). *In vitro* selection of RNA molecules that bind specific ligands. Nature.

[22] Ng EW (2006). Pegaptanib, a targeted anti-VEGF aptamer for ocular vascular disease. Nat. Rev. Drug. Discov..

[23] Mayer G (2010). Fluorescence-activated cell sorting for aptamer SELEX with cell mixtures. Nat. Protoc..

[24] Pinheiro VB (2012). Synthetic genetic polymers capable of heredity and evolution. Science.

[25] Sefah K (2014). *In vitro* selection with artificial expanded genetic information systems. Proc. Natl Acad. Sci. USA.

[26] Spill F (2016). Controlling uncertainty in aptamer selection. Proc. Natl Acad. Sci. USA.

[27] Pfeiffer F (2018). Identification and characterization of nucleobase-modified aptamers by click-SELEX. Nat. Protoc..

[28] Wang J (2017). Multiparameter Particle Display (MPPD): A Quantitative Screening Method for the Discovery of Highly Specific Aptamers. Angew. Chem. Int. Ed. Engl..

[29] McKeague M (2015). Analysis of *In Vitro* Aptamer Selection Parameters. J. Mol. Evol..

[30] Ruscito A (2017). *In Vitro* Selection and Characterization of DNA Aptamers to a Small Molecule Target. Curr. Protoc. Chem. Biol..

[31] Fitter S, James R (2005). Deconvolution of a complex target using DNA aptamers. J. Biol. Chem..

[32] Schutze T (2011). Probing the SELEX process with next-generation sequencing. PLoS One.

[33] Liang C (2015). Comparison of the methods for generating single-stranded DNA in SELEX. Analyst.

[34] Avci-Adali M, Paul A, Wilhelm N, Ziemer G, Wendel HP (2009). Upgrading SELEX technology by using lambda exonuclease digestion for single-stranded DNA generation. Molecules.

[35] Paul A, Avci-Adali M, Ziemer G, Wendel HP (2009). Streptavidin-coated magnetic beads for DNA strand separation implicate a multitude of problems during cell-SELEX. Oligonucleotides.

[36] Svobodova M, Pinto A, Nadal P, CK OS (2012). Comparison of different methods for generation of single-stranded DNA for SELEX processes. Anal. Bioanal. Chem..

[37] Marimuthu C, Tang TH, Tominaga J, Tan SC, Gopinath SC (2012). Single-stranded DNA (ssDNA) production in DNA aptamer generation. Analyst.

[38] Famulok M, Mayer G (2014). Aptamers and SELEX in Chemistry & Biology. Chem. Biol..

[39] Kilili GK, Tilton L, Karbiwnyk CM (2016). [Letter to the Editor] NaOH concentration and streptavidin bead type are key factors for optimal DNA aptamer strand separation and isolation. Biotechniques.

[40] Cruz-Toledo J (2012). Aptamer Base: a collaborative knowledge base to describe aptamers and SELEX experiments. Database.

[41] Le ATH, Krylova SM, Kanoatov M, Desai S, Krylov SN (2019). Ideal-Filter Capillary Electrophoresis (IFCE) Facilitates the One-Step Selection of Aptamers. Angew. Chem. Int. Ed. Engl..

[42] Zong C, Liu J (2019). The Arsenic-Binding Aptamer Cannot Bind Arsenic: Critical Evaluation of Aptamer Selection and Binding. Anal. Chem..

[43] Valenzano S (2016). Screening and Identification of DNA Aptamers to Tyramine Using *in Vitro* Selection and High-Throughput Sequencing. ACS Comb. Sci..

[44] McKeague M (2015). Comprehensive analytical comparison of strategies used for small molecule aptamer evaluation. Anal. Chem..

[45] Lau CK (2015). Structural conservation despite huge sequence diversity allows EPCR binding by the PfEMP1 family implicated in severe childhood malaria. Cell Host Microbe.

[46] Tan J (2016). A LAIR1 insertion generates broadly reactive antibodies against malaria variant antigens. Nature.

[47] Deitsch KW, Dzikowski R (2017). Variant Gene Expression and Antigenic Variation by Malaria Parasites. Annu. Rev. Microbiol..

[48] Kushwaha A, Takamura Y, Nishigaki K, Biyani M (2019). Competitive non-SELEX for the selective and rapid enrichment of DNA aptamers and its use in electrochemical aptasensor. Sci. Rep..

[49] Dunn, M. R., Jimenez, R. M. & Chaput, J. C. Analysis of aptamer discovery and technology. *Nature Reviews Chemistry* **1** (2017).

[50] Ozer A, Pagano JM, Lis JT (2014). New Technologies Provide Quantum Changes in the Scale, Speed, and Success of SELEX Methods and Aptamer Characterization. Mol. Ther. Nucleic Acids.

[51] Shangguan D, Cao ZC, Li Y, Tan W (2007). Aptamers evolved from cultured cancer cells reveal molecular differences of cancer cells in patient samples. Clin. Chem..

[52] Alam KK (2018). Poly-Target Selection Identifies Broad-Spectrum RNA Aptamers. Mol. Ther. Nucleic Acids.

[53] Song MY, Nguyen D, Hong SW, Kim BC (2017). Broadly reactive aptamers targeting bacteria belonging to different genera using a sequential toggle cell-SELEX. Sci. Rep..

[54] Tham WH (2010). Complement receptor 1 is the host erythrocyte receptor for Plasmodium falciparum PfRh4 invasion ligand. Proc. Natl Acad. Sci. USA.

[55] Li M (2008). Selecting aptamers for a glycoprotein through the incorporation of the boronic acid moiety. J. Am. Chem. Soc..

[56] Mayer DC (2009). Glycophorin B is the erythrocyte receptor of Plasmodium falciparum erythrocyte-binding ligand, EBL-1. Proc. Natl Acad. Sci. USA.

[57] Lim, C., Dankwa, S., Paul, A. S. & Duraisingh, M. T. Host Cell Tropism and Adaptation of Blood-Stage Malaria Parasites: Challenges for Malaria Elimination. *Cold Spring Harb Perspect Med* **7**, 10.1101/cshperspect.a025494 (2017). 10.1101/cshperspect.a025494 PMC5666624 28213436

[58] Joseph DF (2019). DNA aptamers for the recognition of HMGB1 from Plasmodium falciparum. PLoS One.

[59] Frith KA (2018). Towards development of aptamers that specifically bind to lactate dehydrogenase of Plasmodium falciparum through epitopic targeting. Malar. J..

[60] Jain P, Chakma B, Singh NK, Patra S, Goswami P (2016). Aromatic Surfactant as Aggregating Agent for Aptamer-Gold Nanoparticle-Based Detection of Plasmodium Lactate Dehydrogenase. Mol. Biotechnol..

[61] Choi SJ, Ban C (2016). Crystal structure of a DNA aptamer bound to PvLDH elucidates novel single-stranded DNA structural elements for folding and recognition. Sci. Rep..

[62] Geldert A (2017). Kenry & Lim, C. T. Paper-based MoS2 nanosheet-mediated FRET aptasensor for rapid malaria diagnosis. Sci. Rep..

[63] Godonoga M (2016). A DNA aptamer recognising a malaria protein biomarker can function as part of a DNA origami assembly. Sci. Rep..

[64] Dirkzwager RM, Kinghorn AB, Richards JS, Tanner JA (2015). APTEC: aptamer-tethered enzyme capture as a novel rapid diagnostic test for malaria. Chem. Commun..

[65] Cherney LT, Obrecht NM, Krylov SN (2013). Theoretical modeling of masking DNA application in aptamer-facilitated biomarker discovery. Anal. Chem..

[66] Berezovski MV, Lechmann M, Musheev MU, Mak TW, Krylov SN (2008). Aptamer-facilitated biomarker discovery (AptaBiD). J. Am. Chem. Soc..

[67] Trager W, Jensen JB (1976). Human malaria parasites in continuous culture. Science.

[68] Amaratunga C (2012). Artemisinin-resistant Plasmodium falciparum in Pursat province, western Cambodia: a parasite clearance rate study. Lancet Infect. Dis..

[69] Lopera-Mesa TM (2013). Plasmodium falciparum clearance rates in response to artesunate in Malian children with malaria: effect of acquired immunity. J. Infect. Dis..

[70] Shangguan D (2008). Identification of liver cancer-specific aptamers using whole live cells. Anal. Chem..

[71] R: A language and environment for statistical computing R Found Stat Comput:Vienna, Austria (2011).

