# Supplementary material for: High-efficiency enrichment enables identification of aptamers to circulating Plasmodium falciparum-infected erythrocytes
Source: Sci Rep. 2020 Jun 16;10:9706. doi: 10.1038/s41598-020-66537-1 (PMC7298056; doi:10.1038/s41598-020-66537-1)
Supplement: Supplementary file 1 — Supplementary information. [file 41598_2020_66537_MOESM1_ESM.pdf]

Supplementary Information

High-efficiency enrichment enables identification of aptamers to circulating *Plasmodium falciparum*-infected erythrocytes

Eugene K. Oteng[1*], Wenjuan Gu[2], Maureen McKeague[3,4]

[1]Laboratory of Malaria and Vector Research, National Institute of Allergy and Infectious Diseases, National Institutes of Health, Rockville, Maryland, 20852 USA.

[2] Clinical Research Directorate/Clinical Monitoring Research Program, Leidos Biomedical Research, Inc., Frederick National Laboratory for Cancer Research, Frederick, Maryland, 21702 USA.

[3] Department of Pharmacology and Therapeutics, McGill University, 3655 Prom. Sir-William-Osler, Montreal, Quebec H3G 1Y6, Canada

[4] Department of Chemistry, McGill University, 801 Sherbrooke Street West, Montreal, Quebec H3A 0B8, Canada

p. 2 Table S1. Sequences of selected aptamers.

p. 3 Table S2. Profile of literature analyzed.

p. 4 Table S3. Univariate comparison of methods used in 43 SELEX publications by number of rounds.

p. 5 Figure S1. Box plot comparing the estimated number of sequences in the initial library (i.e. library size) for selection experiments incorporating alkaline treatment or other single-strand generating methods.

p. 6 Figure S2. Box plot comparing size of the random region in the library used for selection experiments incorporating alkaline treatment or other single-strand generating methods.

Note: Supplementary files with the reads from alkaline and lambda SELEX are available online.

Supplementary Table S4 contains curated data from the Aptamer Base and from the literature as described in Table S2. Available online as a downloadable .xlsx spreadsheet.

	Grouping	Sequences of random region	Read Count*
Lambda			
	Malaria.1	CCAAACGCGCTGGTTTGGAATGCTTAGAGTTTGATCGATGCCTAA	40640
	Malaria.2	GTGCAGACAGCTAGTAGTGGTGACTTTCTGTCCAAATCAAGATAA	1786
	Malaria.3	CACTAGGATCTAGGGAAAAATCTCGTAAATACTATACTCCTGAGC	938
	--	ACGTTTTAGCTACACGTGCGGGCAGTACCCTCACTGGGAAACAAC	444
	--	ATAGCCCGACTATATCGGCAATACACTGTGCTGGATCTTCAGGGT	326
	--	TAGCTGAGTTGTACTCGGATGTGTAGGTTTTTAGGCTAGTAATCA	271
	--	ATAAAAAGCAGGTGGCCTTGTGATTGGATTTATATTGAAAGTACA	261
	--	GTGCAGACAGCTAGTAGTGGTGACTTTCTGTCCAAATCAAGATGA	243
	--	GTGCGGACAGCTAGTAGTGGTGACTTTCTGTCCAAATCAAGATAA	228
	--	CCGCTGCAGACGCGCAAGTGAGATCTGTTCTTTATTGTATG	227
Alkaline			
	Malaria.3	CACTAGGATCTAGGGAAAAATCTCGTAAATACTATACTCCTGAGC	37
	--	TAATGGTGTATTGTTGACTGTTCGCATCGCCTTTAGCTCCGAGCC	16
	--	CAAGTAGAACGATCAAAATAAGATAAAAAGCCTTTTTAAAGCTTA	15
	--	AAGATGAGGCATTTGTATCGATAAAACCGCGTATGACGGTTCCTA	15
	--	TTAGCCTGGCCAAGTTTGTTGTAAATGTGATAATTTCGTTCGAGC	11
	--	CGCGGAAATATCGAATAATGAGGATGAGTGAGTTAAGATGGTGAA	11
	--	GCAGGGTGCTTTAACGTGCCTATACGTTCGTATCGCCTTATCTTT	11
	--	AGGTAACTTGCGGTCAATAGAAGGGTGGATTAATGTACGATCTCA	11
	--	TGCGTGGTTGCCTAGGCTCTAGGGAACCCTAGTCTCATTATATGC	11
	--	TTCGCTCACGGGGTCTACGCGTACCGGACAATTTTGCTTCACATG	10

*Total reads analyzed from lambda and alkaline SELEX were 378,439 and 965,748 respectively

Table S1. Sequences of selected aptamers.

Table S2. Profile of literature analyzed.

No. of Papers	Condition
492	All SELEX experiments from 1990 to 2013 reporting K_d values (Ref: http://www.ncbi.nlm.nih.gov/pubmed/26530075)
	325 papers performing SELEX with RNA, dsDNA, or modified nucleic acids
	23 papers with incomplete data (e.g. did not report the number of rounds or single-strand recovery method)
	41 papers using Capillary Electrophoresis-SELEX, Automated SELEX, Non-SELEX or Microfluidic SELEX
	42 papers that selected for small molecule targets
	1 paper retracted
	17 papers describing SELEX experiments of intermediate length (11-14 rounds)
43	Analyzed

Table S3. Univariate comparison of methods used in 43 SELEX publications by number of rounds.

Characteristics	Efficient (≤10 rounds) N=30	Inefficient (≥15 rounds) N=13	p-value*
Alkaline use	9 (30.0%)	12 (92.3%)	0.0002
Average K_d (M) : mean (range)	3.4e-07 (2.1e-11 – 5.5e-06)	4.3e-07 (1.3e-08 - 2.9e-06)	0.6061
Target type			0.0188
Cells	2 (6.7%)	5 (38.5%)	
Protein	22 (73.3%)	8 (61.5%)	
Other	6 (20.0%)	0 (0.0%)	
Partitioning method			0.0053
Affinity chromatography	21 (70%)	5 (38.5%)	
Centrifugation and cell wash	1 (3.3%)	6 (46.2%)	
Filtration	6 (20.0%)	2 (15.4%)	
Year (range)	2007 (1992-2001)	2006 (1990-2011)	0.9468

* Fisher's exact test for categorical variables; Wilcoxon rank sum test for numerical variables

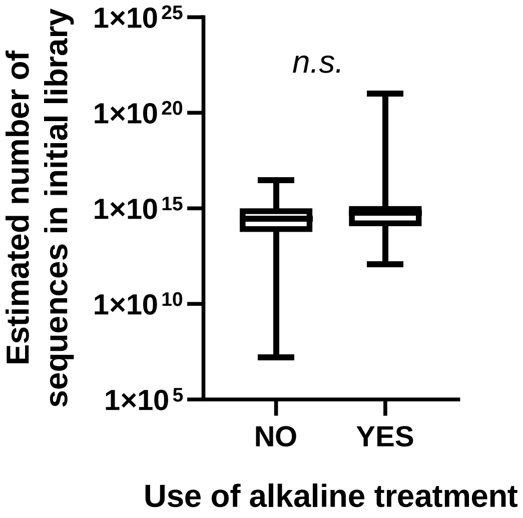

Figure S1. Box plot comparing the estimated number of sequences in the initial library (i.e. library size) for selection experiments incorporating alkaline treatment or other single-strand generating methods. Data was curated from the literature as described in Table S2. Values for each experiment are available in Table S4. Most publications did not provide the estimates, so we calculated the number of sequences based on the experimental conditions. No significant difference was observed.

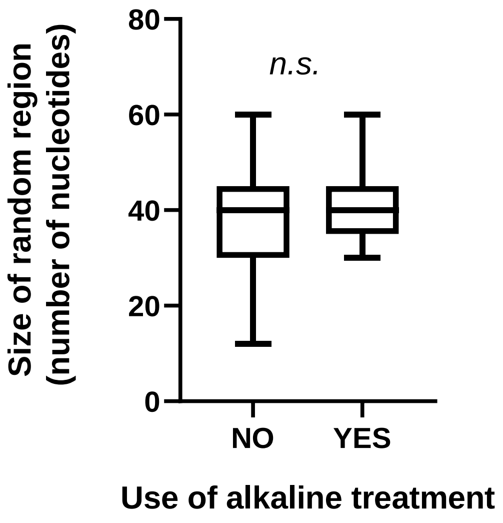

Figure S2. Box plot comparing size of the random region in the library used for selection experiments incorporating alkaline treatment or other single-strand generating methods. Data was curated from the literature as described in Table S2. Values for each experiment are available in Table S4. No significant difference was observed.
